# BMI1 inhibits senescence and enhances the immunomodulatory properties of human mesenchymal stem cells via the direct suppression of MKP-1/DUSP1

**DOI:** 10.18632/aging.101000

**Published:** 2016-07-22

**Authors:** Jin Young Lee, Kyung-Rok Yu, Hyung-Sik Kim, Insung Kang, Jae-Jun Kim, Byung-Chul Lee, Soon Won Choi, Ji-Hee Shin, Yoojin Seo, Kyung-Sun Kang

**Affiliations:** ^1^ Adult Stem Cell Research Center, College of Veterinary Medicine, Seoul National University, Seoul 151-742, South Korea; ^2^ Research Institute for Veterinary Medicine, College of Veterinary Medicine, Seoul National University, Seoul 151-742, Korea; ^3^ Hematology Branch, National Heart, Lung and Blood Institute, National Institutes of Health, Bethesda, MD 20892, USA; ^4^ Pusan National University School of Medicine, Busan 49241, South Korea; ^5^ Biomedical Research Institute, Pusan National University Hospital, Busan 49241, South Korea

**Keywords:** hMSCs, Hypoxia, BMI1, immunomodulation, aging, MKP-1

## Abstract

For the application of mesenchymal stem cells (MSCs) as clinical therapeutics, the regulation of cellular aging is important to protect hMSCs from an age-associated decline in their function. In this study, we evaluated the effects of hypoxia on cellular senescence and the immunomodulatory abilities of hUCB-MSCs. Hypoxic-cultured hUCB-MSCs showed enhanced proliferation and had increased immunosuppressive effects on mitogen-induced mononuclear cell proliferation. We found that BMI1, a member of the polycomb repressive complex protein group, showed increased expression in hypoxic-cultured hUCB-MSCs, and the further knock-down of BMI1 in hypoxic cells induced decreased proliferative and immunomodulatory abilities in hUCB-MSCs, along with COX-2/PGE_2_ down-regulation. Furthermore, the expression of phosphorylated p38 MAP kinase increased in response to the over-expression of BMI1 in normoxic conditions, suggesting that BMI1 regulates the immunomodulatory properties of hUCB-MSCs via p38 MAP kinase-mediated COX-2 expression. More importantly, we identified BMI1 as a direct repressor of MAP kinase phosphatase-1 (MKP-1)/DUSP1, which suppresses p38 MAP kinase activity. In conclusion, our results demonstrate that BMI1 plays a key role in the regulation of the immunomodulatory properties of hUCB-MSCs, and we suggest that these findings might provide a strategy to enhance the functionality of hUCB-MSCs for use in therapeutic applications.

## INTRODUCTION

Mesenchymal stem cells are a promising source for cell therapy of inflammatory diseases because of their profound immunomodulatory abilities, which include effects on the proliferation and function of immune cells through their secretory molecules [1, 2]. Due to their potent immunosuppressive properties, MSCs have been used for treating acute and chronic inflammatory disorders, including graft-versus-host diseases and atopic dermatitis [3-5]. However, previous studies have reported that MSCs enter senescence and show chromosomal aberrations during long-term cultures for the expansion of the cell population [6-8]. Furthermore, our group previously reported that replicatively senescent MSCs lose their therapeutic efficacies because of decreased immunomodulatory cytokine production [9]. In this context, the effect of senescence in attempts to expand the number of cells is a critical factor in regulating the quality as well as the quantity of MSCs available for the therapeutic applications.

Because the partial pressure of oxygen changes as blood circulates through the circulatory system, adult tissues experience differences in the distribution of oxygen tensions [10]. MSCs reside in their niche *in vivo*, where the oxygen levels is relatively lower (1∼8%) than it is in the atmosphere of regular cell cultures (20%) [11]. Also, in clinical applications, MSCs are transplanted into the injured sites, where the oxygen tension is low. Low oxygen levels during the *in vitro* culture of MSCs enhances proliferation and early chondrogenic differentiation and diminishes osteogenesis/adipogenesis [12, 13]. Recent evidence suggests that low-oxygen environments have beneficial effects on protecting stem cells from cellular senescence [14-16]. Furthermore, several groups have reported that hypoxic pre-conditioning enhances the therapeutic efficacies of MSCs in treating ischemic injuries by inducing metabolic changes and by facilitating vascular cell mobilization and skeletal muscle fiber regeneration [16, 17].

One of the prominent immunomodulatory factors of MSCs is prostaglandin E_2_ (PGE_2_), which is synthesized from arachidonic acid catalyzed by cyclooxygenase-1 and cyclooxygenase-2 [18]. COX-2 is a key enzyme for producing PGE_2_ in response to inflammatory stimuli [19], and it has been investigated as a therapeutic target to alleviate excess inflammatory responses [20, 21]. Our previous studies explored the mechanism by which COX-2/PGE_2_ expression is regulated via the phosphorylation of p38 MAP kinase in response to inflammatory stimuli in human umbilical cord blood-derived MSCs (hUCB-MSCs) [9]. MAP kinase phosphatase (MKP)-1, also referred to as dual-specific phosphatase 1 (DUSP1), has been reported to decrease COX-2 expression through the suppression of the p38 MAP kinase pathway [22-24]. However, the regulatory mechanisms by which MKP-1 controls the immuno-suppressive properties of MSCs remain to be determined.

BMI1 is a member of the polycomb repressive complex (PRC) protein group that plays pivotal roles in maintaining the ability for self-renewal and proliferation in various types of stem cells. PRCs suppress target genes through modifying the methylation and ubiquitination of histones [25, 26]. BMI1 in particular has been reported to regulate cellular senescence and proliferation via the repression of the INK4A-ARF locus, which encodes the tumor suppressor p16^INK4a^ [27, 28]. Mice deficient in Bmi1 show premature senescence and a decreased life span, as well as a loss of mitochondrial function accompanied by increased reactive oxygen species (ROS) levels and the activation of DNA damage responses [29, 30]. Although the up-regulation of BMI1 expression in hypoxia via the cooperative transactivation of hypoxia-inducible factor-1 α (HIF-1 α) and Twist has been reported [31], the role of BMI1 in regulating the therapeutic properties of hMSCs has not been elucidated.

In the present study, we assessed the effects of BMI1-induced senescence on the immunomodulatory functions of hUCB-MSCs and investigated the underlying mechanisms. Our study provides evidence that BMI1 expression levels are maintained following consecutive passages in hypoxia, and the regulation of BMI1 gene expression alters immunosuppressive functions by suppressing MKP-1, a major negative regulator of p38 MAP kinase in hUCB-MSCs. Our results highlight the advantages of hypoxic cultures for hUCB-MSCs, revealing a novel mechanism by which BMI1 regulates the immune response of hUCB-MSCs.

## RESULTS

### Hypoxic culturing decreases cellular senescence in hUCB-MSCs with increased BMI1 expression

**Figure 1:**
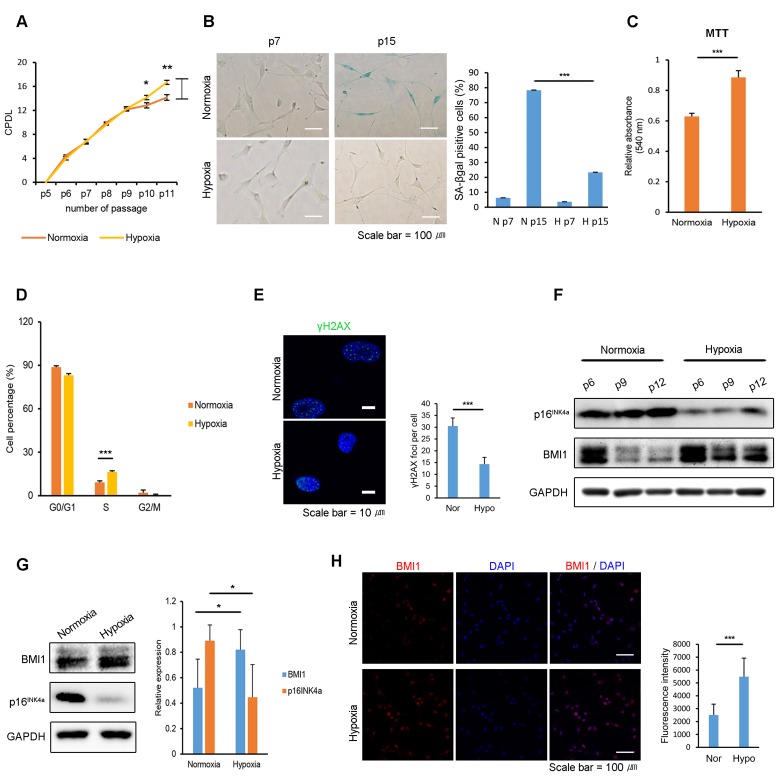
Hypoxia protects hUCB-MSCs from cellular senescence and increases BMI1 expression (**A**) Cumulative population doubling levels were measured to investigate the effect of hypoxic culturing on the proliferation of hUCB-MSCs. At each passage, the same numbers of cells were seeded and cultured in normoxic (20% O_2_) or hypoxic (1% O_2_) conditions. After 4 days, cells were harvested and counted with a hemocytometer. (n=3) (**B**) SA-beta galactosidase staining was conducted in early and late passages of normoxic- and hypoxic-cultured hUCB-MSCs. (**C**) MTT assay was conducted to assess the proliferation of normoxic- and hypoxic-cultured MSCs (n=3). (**D**) Cell cycle distribution was analyzed with propidium iodide using flow cytometry (n=3). (**E**) Immunostaining of γH2AX was performed in the passaged hUCB-MSCs in normoxia and hypoxia. Each images show the representative and the graph indicates the quantification of loci per cell. (**F**) Protein expression of BMI1 and p16^INK4a^ was determined via western blot analysis. (**G**) Western blotting was performed to evaluate the expression of BMI1 and p16^INK4a^ in hUCB-MSCs cultured for three days in normoxia or hypoxia. Average BMI1 and p16^INK4a^ band intensities of three independent replicate experiments were quantified and the representative immunoblots are shown. (**H**) Expression of BMI1 in normoxic- and hypoxic-cultured hUCB-MSCs was determined via immunocytochemistry. Representative images from at least three independent experiments are shown. Error bars represent mean±s.e.m. from three separate experiments. * *P*<0.05, ** *P*<0.01, *** *P*<0.005 using Student's *t*-test.

It has been reported that combining low cell densities and hypoxic culturing in expanding human bone-marrow-derived MSCs preserves their proliferative capacity without inducing senescence [32]. To determine the effects of a hypoxic environment on the proliferation and cellular senescence of hUCB-MSCs, equal numbers of cells were seeded in normoxic and hypoxic (1% O_2_) cultures. After 4-6 consecutive passages, normoxic-cultured hUCB-MSCs showed a decreased proliferation rate, whereas hypoxic-cultured cells maintained their ability to proliferate (Fig. 1A). Furthermore, hypoxic culture conditions inhibited the senescence-associated Δ-galactosidase (SA-Δ-gal) activity of the hUCB-MSCs compared to the activity in normoxic conditions (Fig. 1B). The increased proliferative ability of hypoxic-cultured hUCB-MSCs was confirmed via 3-(4,5-dimethylthiazol-2-yl)-2,5-diphenyltetrazolium bromide (MTT) assay and a cell cycle analysis using propidium iodide staining (Fig. 1C-D). Hypoxic conditions increased the number of cells in the S-phase and decreased the number of cells in the G0/G1 phase. In addition, passaged hUCB-MSCs in hypoxia showed decreased γ-H2AX foci compared to the cells senesced in normoxia (Fig. 1E). It suggests that the hypoxic culture environment suppressed DNA damage response of the hUCB-MSCs. Hypoxic-cultured hUCB-MSCs maintained their characteristic cell surface-marker profile and capability for multi-lineage differentiation (Fig. S1). Western blot analysis showed that a low oxygen environment decreased the expression of p16^INK4a^, a senescence marker, and increased BMI1 in hUCB-MSCs (Fig. 1F-G). The increased expression of BMI1 by hUCB-MSCs cultured in a low oxygen environment was also investigated immunocytochemically (Fig. 1H). Hierarchical clustering indicates that normoxic- and hypoxic-cultured hUCB-MSCs have distinctly altered patterns of gene expression for processes related to cellular senescence, such as cell growth, cell cycle control and the p53 pathways (Fig. S2). Taken together, these results show that hypoxic culturing prevents hUCB-MSCs from undergoing cellular senescence in association with increased expression levels of BMI1.

### Hypoxia-induced BMI1 regulates cellular senescence in hUCB-MSCs

**Figure 2:**
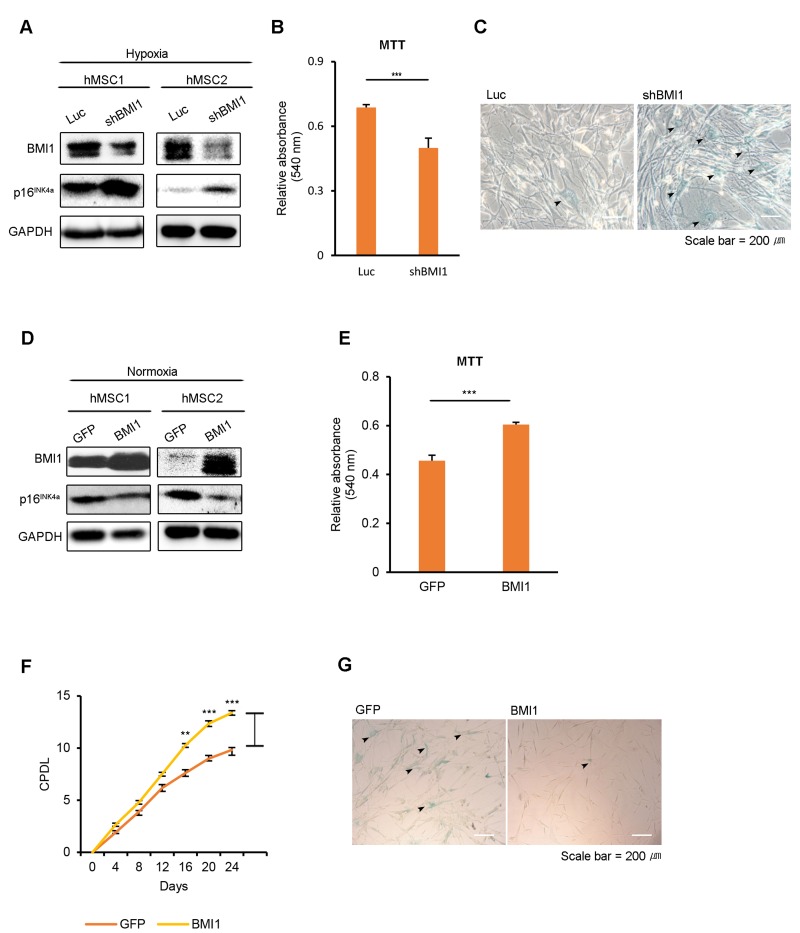
BMI1 regulates cellular senescence in hUCB-MSCs (**A**-**C**) To assess the role of BMI1 in hypoxia, hypoxic-cultured hUCB-MSCs were transfected with Luc control and shBMI1, and the senescence phenotype was investigated. (A) Expression levels of BMI1 and p16^INK4a^ proteins were confirmed via western blot analysis. (**B**) To assess the proliferation of BMI1-down-regulated hUCB-MSCs, an MTT assay was performed. (**C**) The senescent state of cells was confirmed via SA-β-gal staining. (**D**-**G**) To confirm the effects of BMI1 on cellular senescence, normoxic-cultured hUCB-MSCs were induced to overexpress GFP and BMI1. (**D**) Western blot analysis was performed to confirm the expression levels of BMI1 and p16INK4a proteins. (**E**) MTT assay was conducted to evaluate the proliferation of BMI1-up-regulated hUCB-MSCs. (**F**) After the overexpression of BMI1, CPDLs were determined to evaluate the proliferative ability of the hUCB-MSCs. (**G**) After several passages, the senescent state of cells was confirmed via SA-β-gal staining. The results show 1 representative of 3 independent experiments. Error bars represent mean±s.e.m. from three separate experiments. ** *P*<0.01, *** *P*<0.005 using Student's *t*-test.

To elucidate the role of BMI1 in hypoxic-cultured hUCB-MSCs, we conducted BMI1 loss-of-function studies using retroviral shRNA. First, we assessed the senescence phenotype of BMI1-down-regulated hUCB-MSCs compared to control Luc-hUCB-MSCs in hypoxia. The depletion of BMI1 increased SA-Δ-gal staining and p16^INK4a^ expression, showing the accelerated aging of these hUCB-MSCs (Fig. 2A, C). Cellular proliferation was also decreased in shBMI1-MSCs (Fig. 2B). To explore the role of increased BMI1 expression in hypoxic-cultured hUCB-MSCs, we overexpressed BMI1 in normoxic conditions and examined the effects this had on cellular senescence. To investigate the effect of BMI1 in regulating proliferation and senescence, hUCB-MSCs were transduced with control GFP or BMI1 viral vectors. The overexpression of BMI1 and the decreased expression of p16^INK4a^ was confirmed via western blotting (Fig. 2D). BMI1-transduced hUCB-MSCs maintained their proliferative capacity, whereas GFP-transduced cells showed decreased proliferation following consecutive passages (Fig. 2E, F). BMI1-overexpressing hUCB-MSCs showed a reduced SA-Δ-gal activity compared to that of the control GFP-transduced cells, which showed an elevated SA-Δ-gal activity along with a flattened or lengthened morphology (Fig. 2G). In our previous studies, a decreased expression of histone deacetylases (HDACs), followed by the downregulation of polycomb group genes (PcGs), such as BMI1, EZH2 and SUZ12, was observed in senescent hUCB-MSCs. Therefore, we assessed the effects of other polycomb group proteins, such as EZH2 and SUZ12, on the hUCB-MSC senescence phenotype. BMI1-transduced cells maintained their proliferative ability, whereas EZH2-, SUZ12- or GFP-transduced hUCB-MSCs showed a decrease in proliferation following consecutive passages (Fig. S3A). In line with the proliferation assay data, BMI1 showed most significantly reduced SA-Δ-gal activity after 6-8 passages (Fig. S3B). These results revealed that BMI1 regulates the cellular senescence of hUCB-MSCs in a hypoxic environment.

### BMI1 regulates the immunosuppressive properties of hUCB-MSCs in hypoxia

**Figure 3:**
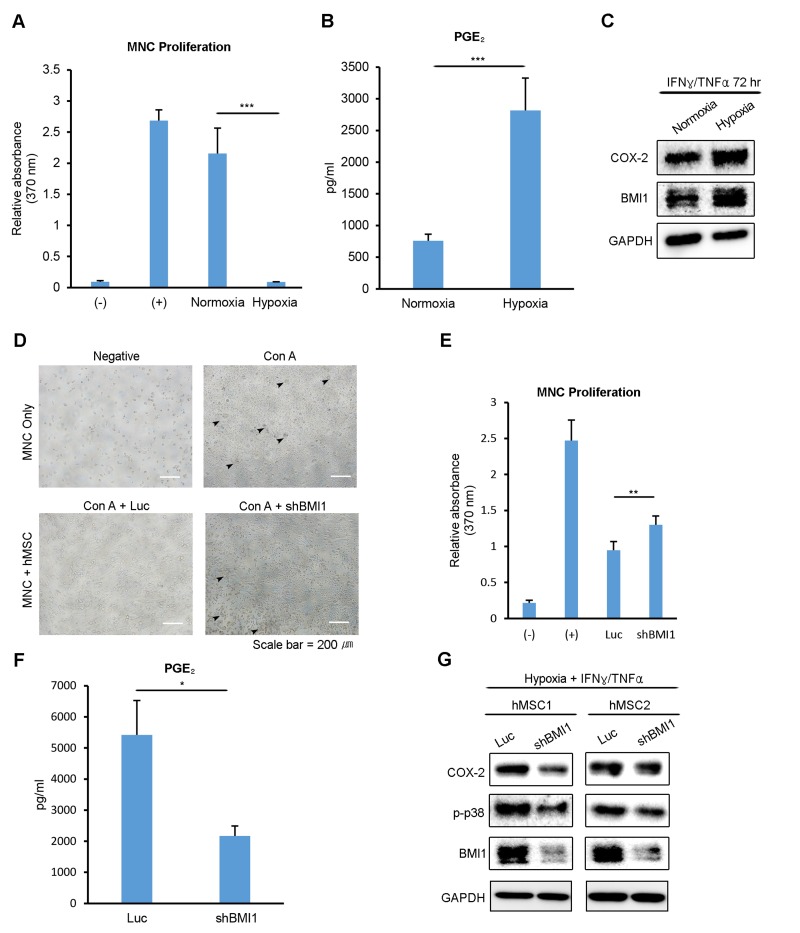
Low oxygen environment increases the BMI1-dependent immunomodulatory properties of hUCB-MSCs (**A**, **B**) The culture supernatant from normoxic- and hypoxic-cultured hUCB-MSCs was collected after treatment with IFN-γ and TNF-α for 72 hours. (**A**) hUCB-MNCs were cultured with each supernatant after being activated with concanavalin A. After 3 days, the proliferation of MNCs was determined using a BrdU assay kit. (**B**) PGE_2_ concentration was measured using an ELISA kit. (**C**) Proteins were extracted after collecting the supernatant, and a western blot analysis was performed with the indicated antibodies. Representative blots are shown. (**D**) The effect on the immunosuppressive properties of BMI1-down-regulated hUCB-MSCs was investigated via a co-culture experiment. (**E**) Proliferation levels of hUCB-MNCs were measured using a BrdU assay kit. (**F**) PGE_2_ concentration levels were measured from the culture supernatant of control- and shBMI1-induced hUCB-MSCs. (**G**) Western blot analysis of COX-2 signaling factors was conducted. Error bars represent mean±s.e.m. from three separate experiments. * *P*<0.05, ** *P*<0.01, *** *P*<0.005 using Student's *t*-test.

Our previous study revealed that replicatively senescent hUCB-MSCs have compromised immunomodulatory functions, with decreased PGE2 levels. To determine whether the immunomodulatory functions of hUCB-MSCs are related to the oxygen tension levels during culturing, we tested the inhibitory effect of normoxic- and hypoxic-cultured hUCB-MSCs on the proliferation of mitogen (concanavalin A)-induced human umbilical cord blood-derived mononuclear cells (hUCB-MNCs). Normoxic- and hypoxic-cultured hUCB-MSCs were treated with IFN-γ and TNF-α for 72 hours, and hUCB-MNCs were treated with the collected supernatant. hUCB-MNC proliferation was suppressed to a greater extent in the presence of the supernatant from hypoxic-cultured hUCB-MSCs (Fig. 3A). In addition, the PGE_2_ level in the cell culture media was notably higher in the supernatant from the hypoxic-cultured hUCB-MSCs (Fig. 3B). After collecting the supernatant, proteins were extracted from the MSCs and analyzed via western blot. Consistent with the PGE_2_ level, the expression of COX-2, a key enzyme in the production of PGE2, was highly increased in hypoxic-cultured hUCB-MSCs (Fig. 3C). To assess the impact of BMI1 down-regulation on the immunomodulatory properties of hUCB-MSCs, we co-cultured hUCB-MNCs with BMI1-down-regulated hUCB-MSCs after they had been activated by Con A in hypoxia (Fig. 3D). The proliferation of hUCB-MNCs co-cultured with shBMI1-MSCs was increased compared to that of the control MSCs, suggesting a decline in the immunosuppressive function of BMI1-down-regulated hUCB-MSCs (Fig. 3E). In addition, the secretion of PGE_2_ was significantly down-regulated in shBMI1-MSCs (Fig. 3F). To confirm the regulatory effect of BMI1 on the activation of p38 MAP kinase in cells cultured in hypoxia, we compared the phosphorylation of p38 MAP kinase in hypoxic-cultured hUCB-MSCs and BMI1-down-regulated/hypoxic-cultured hUCB-MSCs (Fig. 3G). The expression of phosphorylated p38 MAP kinase was decreased in response to the down-regulation of BMI1 in the hypoxic culture condition (Fig. 3G). These findings suggest that BMI1 regulates immunomodulatory properties via p38 MAP kinase-mediated COX-2 expression in low-oxygen-cultured hUCB-MSCs.

### BMI1 upregulation enhances the immunomodulatory properties of hUCB-MSCs

**Figure 4:**
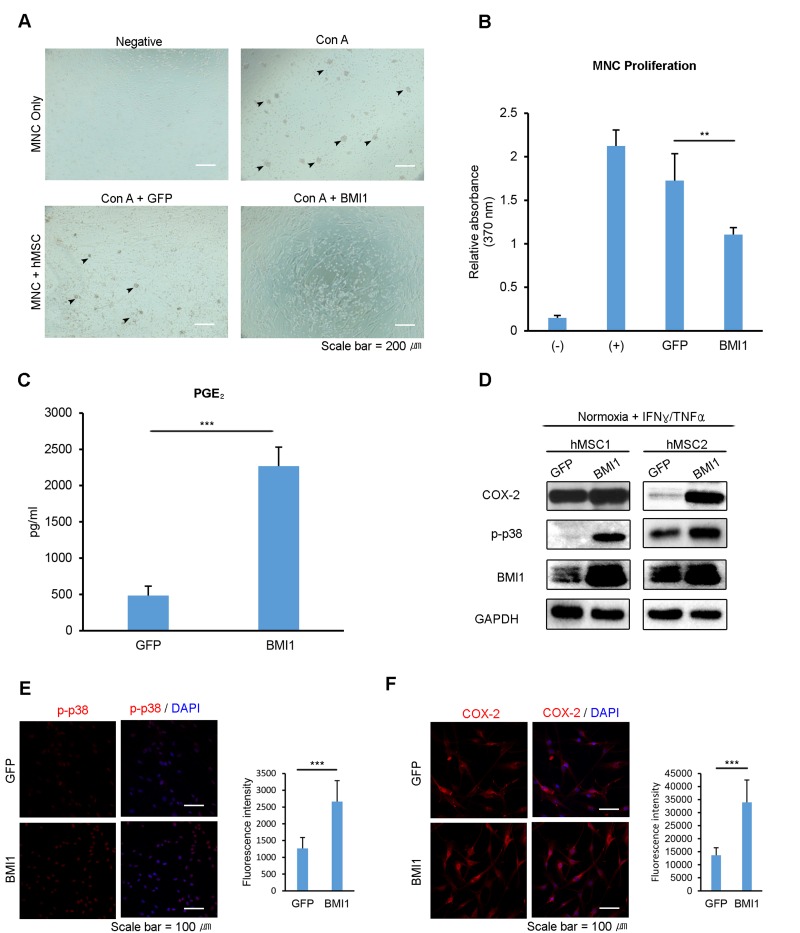
Up-regulation of BMI1 enhances the immunosuppressive effect of hUCB-MSCs To investigate the immunosuppressive properties of BMI1-overexpressing hUCB-MSCs, (**A**) hUCB-MNCs were co-cultured with BMI1-overexpressing hUCB-MSCs and (**B**) the proliferation of hUCB-MNCs was measured with a BrdU proliferation assay kit. (**C**) PGE_2_ concentration was measured in the culture supernatant of GFP- and BMI1-overexpressing hUCB-MSCs. (**D**) COX-2, p-p38 MAP kinase and BMI1 expression levels of BMI1-overexpressing hUCB-MSCs were investigated via western blot analysis after treatment with IFN-γ and TNF-α for 30 minutes. (**E**) Expression of the phosphorylated form of p38 MAP kinase in GFP- and BMI1-up-regulated hUCB-MSCs was determined via immunocytochemistry after treatment with IFN-γ and TNF-α for 30 minutes. The graph shows the fluorescence intensity of p-p38 in each cells. (**F**) COX-2 expression was investigated after treatment with IFN-γ and TNF-α for 24 hours. On the right, the graph indicating the fluorescence intensity of COX-2 is presented. The results show 1 representative of 3 independent experiments. Error bars represent mean±s.e.m. from three separate experiments. Error bars represent mean±s.e.m. from three separate experiments. ** *P*<0.01, *** *P*<0.005 using Student's *t*-test.

To investigate the effect of BMI1 on the immunosuppressive properties of hUCB-MSCs, we co-cultured Con A-activated hUCB-MNCs with BMI1-transduced hUCB-MSCs. The mitogen-induced proliferation of hUCB-MNCs was remarkably reduced when co-cultured with BMI1-overexpressing hUCB-MSCs (Fig 4. A-B). Furthermore, BMI1-overexpressing hUCB-MSCs showed a more than 4-fold increase in the concentration of secreted PGE_2_ (Fig. 4C), suggesting improved immunomodulatory abilities. These data suggest that BMI1 could improve immunomodulatory properties in hUCB-MSCs and protect them from cellular senescence. Our previous study showed that replicatively senescent hUCB-MSCs lose their immunomodulatory abilities because of a decrease in the phosphorylation of p38 MAP kinase [9] and the effect this has on COX-2 expression. To investigate the effects of BMI1 on COX-2 expression and on the phosphorylation of p38 MAP kinase, we performed western blotting with pro-inflammatory cytokine-activated hUCB-MSCs in the presence or absence of BMI1 expression. We found that BMI1 overexpression induced an increase in the phosphorylation of p38 MAP kinase and COX-2 expression, along with a decrease in p16^INK4a^ expression in IFN-γ- and TNF-α-activated hUCB-MSCs (Fig. 4D). In addition, we assessed the effects on other polycomb group proteins on immunomodulating factors, and found that BMI1-overexpressing hUCB-MSCs showed increased COX-2 levels, as demonstrated by western blot analysis (Fig S3C). Increased levels of phosphorylated p38 MAP kinase and COX-2 expression were also confirmed via immunocytochemistry (Fig. 4E-F). These results indicate that BMI1 increases the therapeutic effects of hUCB-MSCs via the up-regulation of COX-2 and the phosphorylation of p38 MAP kinase.

### BMI1 inhibits MKP-1/DUSP1 expression in hypoxia-cultured hUCB-MSCs

**Figure 5:**
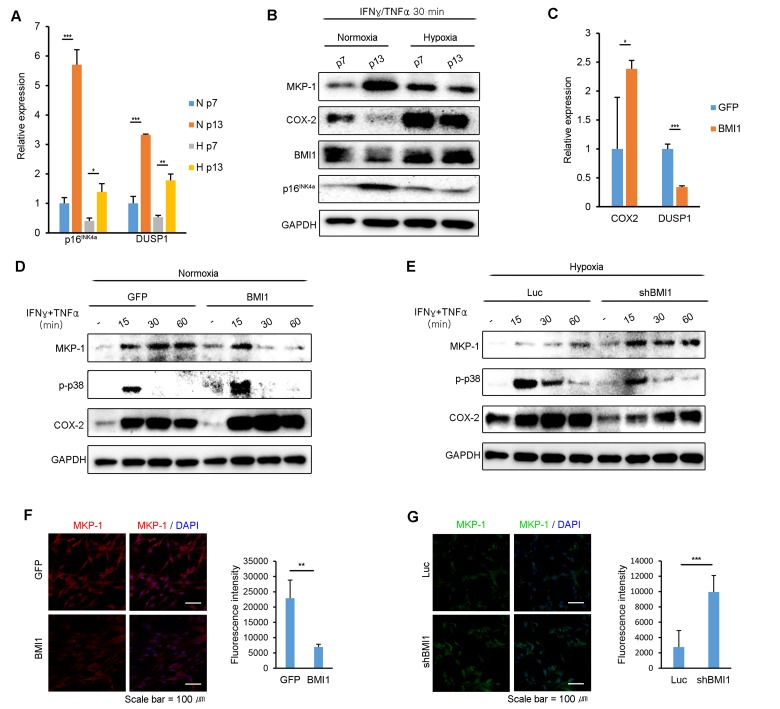
BMI1 negatively regulates DUSP1/MKP-1 in hUCB-MSCs (**A**) DUSP1 and p16^INK4a^ mRNA expression levels were investigated in replicatively senescent hUCB-MSCs in normoxia/hypoxia culture conditions after exposure to IFN-γ and TNF-α for 30 minutes. (**B**) The expression levels of MKP-1, p-p38 and BMI1 in normoxic- or hypoxic-cultured hUCB-MSCs were investigated via western blot analysis in replicatively senescent hUCB-MSCs after treatment with IFN-γ and TNF-α for 30minutes. (**C**) COX-2 and DUSP1 mRNA expression levels were assessed in GFP- and BMI1-overexpressing hUCB-MSCs after activation with IFN-γ and TNF-α for 30 minutes. (**D**) MKP-1 protein expression levels were investigated via western blot analysis in normoxic culture conditions with BMI1-overexpressing hUCB-MSCs. (**E**) MKP-1 protein expression levels were assessed via western blot analysis in hypoxic culture conditions with BMI1-down-regulated hUCB-MSCs. (**F**-**G**) MKP-1 expressions were analyzed by immunocytochemistry in GFP/BMI1 overexpressing and Luc/shBMI1 transfected hUCB-MSCs. The graphs show the fluorescence intensity of MKP-1 in each cells. Error bars represent mean±s.e.m. from three separate experiments. * *P*<0.05, ** *P*<0.01, *** *P*<0.005 using Student's *t*-test.

Because the expression of BMI1 regulates p38 MAP kinase activity in hUCB-MSCs, we examined whether BMI1 also regulates MKP-1/DUSP1 expression, a negative regulator of p38 MAP kinase. As shown in Fig 5A, p16^INK4a^ and DUSP1 mRNA expression highly increased in normoxic culture conditions following consecutive passages compared to in hypoxic culture conditions (Fig. 5A). Furthermore, the protein expression level of MKP-1 was maintained in hUCB-MSCs in hypoxia compared to the level in normoxia following multiple passages after stimulation with IFN-γ and TNF-α (Fig. 5B). To identify whether BMI1 regulates MKP-1/DUSP1 expression in hUCB-MSCs stimulated with IFN-γ and TNF-α, we measured DUSP1 mRNA and MKP-1 protein expression levels in BMI1-up-regulated and BMI1-down-regulated cells. Interestingly, the mRNA expression level of DUSP1 was significantly decreased in BMI1 overexpressing hUCB-MSCs, whereas the COX-2 transcription level was increased (Fig. 5C). In addition, the expression level of MKP-1 and the duration over which it was expressed were both significantly decreased in BMI1-up-regulated hUCB-MSCs, which also showed an increased phosphorylation of p38 MAP kinase and COX-2 expression (Fig. 5D). In contrast, the down-regulation of BMI1 in hypoxic-cultured hUCB-MSCs induced an increase in the expression of MKP-1/DUSP1, along with a decreased phosphorylation of p38 MAP kinase and COX-2 expression (Fig. 5E). Also, we confirmed the expression of MKP-1 in BMI1 overexpressed and down-regulated cells immunocytochemically (Fig. 5F-G). Taken together, these results indicate that BMI1 suppresses DUSP1/MKP-1 expression, which in turn increases p38 MAPK-mediated COX-2 expression.

### BMI1 directly inhibits MKP-1/DUSP1 expression by binding to the promoter of DUSP1

**Figure 6:**
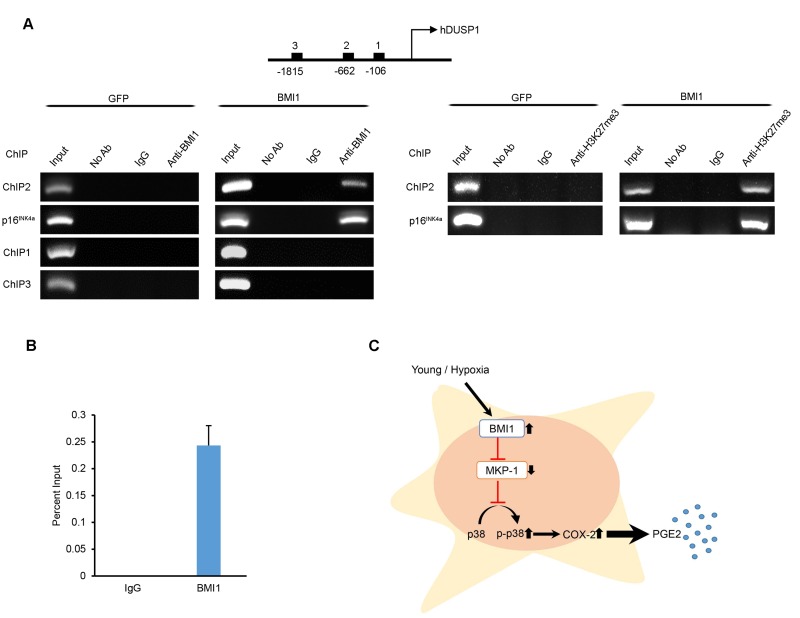
BMI1 directly suppresses DUSP1 by binding to the promoter of DUSP1 (**A**) Chromatin immunoprecipitation was performed to examine BMI1 and H3K27me3 binding to the DUSP1 and p16^INK4a^ promoter of GFP- and BMI1-overexpressing hUCB-MSCs. (**B**) ChIP-quantitative PCR assay results for the DUSP1 promoter region in BMI1-up-regulated hUCB-MSCs. Data represent the means ± SEM (**C**) Schematic representation of the function of BMI1 in hUCB-MSCs in increasing their immunomodulatory properties and protecting the cell from senescence.

To clarify how BMI1 regulates MKP-1/DUSP1 expression, we performed chromatin immuno-precipitation (ChIP) analyses to investigate the direct binding of BMI1 to putative MKP-1/DUSP1 promoter regions. Sequentially located primers (Supplementary Table S1) in the conserved promoter region of DUSP1 were used for immunoprecipitation with the BMI1 antibody. As shown in Figure 6A and 6B, the DUSP1 −662 region within the DUSP1 promoter was enriched with BMI1 and H3K27me3 protein. Furthermore, we confirmed that BMI1 binds specifically to the BMI1 response element of the p16^INK4a^ promoter, as previously reported [33]. A negative control (IgG control) and other putative binding sites (−106, −1815) showed no significant enrichment in the surveyed region. Overall, these findings suggest that BMI1 regulates the immunomodulatory properties of hUCB-MSCs through the direct repression of MKP-1/DUSP1 (Fig. 6C).

## DISCUSSION

In this study, we demonstrate that expanding hUCB-MSCs in a low-oxygen environment has beneficial effects on both cellular senescence and their therapeutic potential in comparison to culturing cells in normoxia. It is well known that hypoxia inhibits senescence and promotes the proliferation of cells, including stem cells and cancer cells [34-36]. The hypoxic culturing of MSCs has been reported to increase their proliferative ability and to extend their cellular lifespan [37], and preculturing human bone-marrow-derived MSCs in hypoxic conditions (1-3% oxygen) improves the speed of their tissue-regenerating effects in a mouse model of ischemia-induced hind-limb injury [38]. However, little is known about how the immunomodulatory functions of MSCs change in hypoxic culture conditions. Rhijn and colleagues demonstrated that the ability of adipose tissue-derived MSCs (ASCs) to inhibit CD4+ and CD8+ T cell proliferation, as well as the expression of the immunomodulatory molecule indoleamine 2,3-dioxygenase (IDO), was not affected by hypoxia [39]. Although the authors found that the inhibition of T cell proliferation was higher at an ASC:PBMC ratio of 1:5 under hypoxia, the underlying mechanism was not analyzed in their study. The possibilities that T cell proliferation is affected by hypoxia or that they are more sensitive to low tryptophan concentrations induced by IDO expression were discussed. However, our study demonstrates that treatment with the supernatant from hypoxic-cultured hUCB-MSCs results in an enhanced immunomodulatory efficacy compared to the effects of treatment with normoxic supernatant and in an elevation in PGE_2_ expression, suggesting that alterations of the concentration of secreted molecules in hypoxia causes enhanced immunosuppressive properties, not changes in T cell sensitivity (Figure 1). Further study may be required to compare the hypoxia-induced alterations of immunosuppressive properties in different sources of MSCs, such as MSCs derived from umbilical cord blood, adipose tissue, or bone marrow.

It has been reported that BMI1 modulates self-renewal and cellular aging by repressing the p16^INK4a^ and p19^Arf^ senescence pathways in hematopoietic stem cells and neural stem cells [40, 41]. Our previous data also demon-strated that polycomb group genes (PcGs), such as BMI1, EZH2, and SUZ12, were down-regulated in senescent MSCs, suggesting that PcGs play an important role in MSC aging. Furthermore, we showed that c-MYC binds to the BMI1 promoter and regulates the expression of BMI1 in early-passage MSCs, which is correlated with hyper-phosphorylated retinoblastoma and activated E2F expression [42]. Du et al. demonstrated that BMI1 expression and transcriptional activation are induced cooperatively by HIF-1 α and Twist under hypoxia [31]. Because c-MYC expression was not significantly increased in hypoxic-cultured human MSCs [43], and multiple mechanisms of counteract activity between HIF-1 and c-MYC have been reported [44, 45], it is possible that hUCB-MSCs might use different pathways to activate BMI1 gene expression in normoxic and hypoxic culture conditions. Therefore, further studies are needed to determine which factors regulate BMI1 expression in normal/senescent or normoxic/hypoxic-cultured hUCB-MSCs.

One important finding in the present study is the identification of BMI1 as a regulator of MSC senescence and immune modulation. In human cord blood-derived hematopoietic CD34+ cells, the enforced expression of BMI1 results in a prolonged maintenance of the stem-cell pool and enhanced self-renewal [46], suggesting that the constitutive expression of BMI1 could regulate adult stem cell functions. In line with this data, we have shown that the retroviral-induced up-regulation of BMI1 in hUCB-MSCs leads to a remarkable delay in senescence and an increase in proliferation rates (Figure 2), indicating a role of BMI1 as an intrinsic regulator of hUCB-MSCs. More importantly, our study provides the first evidence that BMI1 promotes the immunomodulatory function of hUCB-MSCs via the COX-2/PGE_2_ signaling pathway. COX-2 is an inducible rate-limiting enzyme in the PGE_2_ synthesis pathway that mainly regulate PGE_2_ synthesis in inflammatory environments. Our previous report showed that senescent hUCB-MSCs express decreased levels of COX-2/PGE_2_ and phosphorylated p38 MAP kinase [9]. Previous studies have revealed that hypoxia-induced COX-2 expression in endothelial cells and cancer cells regulates angiogenesis and tumor invasiveness [47, 48]. In colorectal tumor cells, HIF-1 α binds a hypoxia-responsive element on the COX-2 promoter, representing implication for colorectal tumor cell survival and angiogenesis [48]. Therefore, the up-regulation of COX-2/PGE_2_ expression by hUCB-MSCs in hypoxic culture conditions is possibly induced by the cooperative transactivation of HIF-1 α and BMI1.

The present data show that hypoxia and BMI1 up-regulation enhance the expression levels of phospho-rylated p38 MAP kinase/COX-2/PGE2. We hypothesized that BMI1 regulates COX-2/PGE2 through the regulation of MKP-1, which is a major suppressor of phosphorylated p38 MAP kinase-mediated COX-2 expression in inflammatory environments [22, 24]. Previous reports have shown that cellular stress increases MKP-1 expression through histone modifications in response to irradiation, arsenite and heat shock [49], whereas hypoxic culture conditions result in decreased MKP-1 activity [50]. In line with these results, our data show that a hypoxic culture environment and the up-regulation of BMI1 induce lower levels of MKP-1 expression compared to that of senescent cells. To determine whether the regulation of MKP-1 expression by BMI1 is due to direct binding, we performed a ChIP assay. We showed that BMI1 specifically bound to one of the putative promoter regions of MKP-1, at position −662 (Figure 4). The novel finding that BMI1 can enhance p38 MAP kinase activity by suppressing the expression of DUSP1 greatly expands our understanding of how BMI1 can influence the immunomodulatory activities of hUCB-MSCs. Further investigations into the other functions of BMI1 via the MKP-1 pathway would be interesting.

In conclusion, our study reveals that hypoxic environments may increase the proliferative and immunomodulatory abilities of hUCB-MSCs. Further-more, we identified BMI1 as a mediator of hypoxia-induced immunosuppressive properties through the direct regulation of the MKP-1/p38 and MAP kinase/COX-2/PGE_2_ pathways. Finally, insight into BMI1-induced immunosuppressive properties and senescence-control mechanisms may provide future possibilities to expand hUCB-MSCs with improved functions for clinically relevant qualities.

## MATERIALS AND METHODS

### Isolation and culture of hUCB-MSCs

For the isolation of hUCB-MSCs, UCB samples were obtained from the umbilical vein immediately after delivery with the informed consent of the mothers. Procedures were approved by the Boramae Hospital Institutional Review Board (IRB) and the Seoul National University IRB (IRB no. E1507/001-011). The hUCB-MSCs were isolated and cultured as previously described [51]. Briefly, the UCB samples were mixed with HetaSep solution (StemCell Technologies, Vancouver, Canada) at a ratio of 5:1 and then incubated at room temperature to deplete the erythrocytes. The supernatant was carefully collected, and mononuclear cells were obtained using Ficoll density-gradient centrifugation at 2500 rpm for 20 minutes. The cells were washed twice in PBS. Cells were seeded at a density of 2×10^5^ to 2×10^6^ cells/cm^2^ on plates in growth medium made from a KSB-3 Complete Medium kit (Kangstem Biotech, Seoul, Korea) and 10% fetal bovine serum (Gibco BRL, USA). After 3 days, the adherent cells formed colonies and the non-adherent cells were removed. For long-term cultures, the cells were seeded at a density of 4×10^5^ cells/10-cm plate, and the cells were subcultured upon reaching 80∼90% confluency. For hypoxic cultures, cells were cultured in an incubator maintained at 5% CO_2_ and 1% O_2_ using the delivery of nitrogen gas (N_2_) from a tank containing pure N_2_ for at least 3 days.

### Characterization of hUCB-MSCs

hUCB-MSCs were triturated into single cells and labeled with monoclonal mouse anti-human fluorochrome-conjugated antibodies: CD29-PE, CD34-FITC, CD45-FITC, CD105-FITC, CD73-PE, and HLA-DR (BD Bioscience, San Jose, USA). The labeled cells were analyzed via flow cytometry using a FACSCalibur system (BD Biosciences).

### In vitro differentiation assay

The in vitro differentiation assay was performed as previously described [52]. For differentiation into osteoblasts and adipocytes, 1×10^5^ cells were plated in 6-well plates. After the cells reached 70-80% confluency, they were treated with an osteogenic differentiation-inducing medium (DMEM containing 10% FBS, 100 nM dexamethasone, 50 μM ascorbic acid 2-phosphate, and 10 mM β-glycero-phosphate) or an adipogenic differentiation-inducing medium (DMEM supplemented with 10% FBS, 200 μM indomethacin, 1 μM dexamethasone, 0.5 mM isobutyl methylxanthine, and 0.5 μg/ml insulin) (all materials from Sigma-Aldrich, St. Louis, USA). The medium was changed every 2-3 days. After 2 weeks of induction, the cells were stained to confirm osteogenic or adipogenic differentiation. To confirm adipogenic differentiation, Oil Red O staining was conducted to detect fat droplets in the differentiated cells. Briefly, cells were fixed with 10% formalin for 1 hour and rinsed with 60% isopropanol before incubation in fresh diluted Oil Red O for 10 minutes. To confirm osteogenic differentiation, Alizarin Red S staining, which is specific for calcium, was performed to detect alkaline phosphate activity. Briefly, cells were rinsed with PBS and fixed with 70% ice-cold ethanol for 1 hour at 4°C. After 3 washes with distilled water, the cells were stained using 40 mM Alizarin Red S (Sigma-Aldrich) for 10 minutes at room temperature.

### Cumulative population doubling level (CPDL)

The proliferation potential of MSCs under each condition (normoxia and hypoxia, regulation of BMI1 expression) was determined by calculating the cumulative population doubling level in continual subculture and growth from a number of cells. At each subculture, the CPDL was calculated from the cell count using the following equation: ln(Nf/Ni)/ln2, where Ni and Nf are initial and final cell count numbers, respectively, and ln is the natural log.

### Senescence-associated beta-galactosidase (SA-Δ-gal) staining

SA-Δ-gal staining was performed as previously described [53]. The hUCB-MSCs were seeded on 6-well plates and incubated for 2-3 days until reaching 70-80% confluency. The cells were washed twice with PBS and fixed with 0.5% glutaraldehyde in PBS (pH 7.2) for 5 minutes at room temperature. The cells were then washed with PBS containing 1 mM MgCl_2_ (pH 7.2) and stained with X-gal solution [1 mg/ml X-gal, 0.12 mM K_3_Fe(CN)_6_, 1 mM MgCl_2_ in PBS at pH 6.0] overnight at 37°C. The cells were washed twice with PBS, and the images were captured using a light microscope (IX70, Olympus, Tokyo, Japan)

### MTT assay

The proliferative potential of the hUCB-MSCs was measured using the 3-(4,5-dimethylthiazol-2-yl)-2,5-diphenyltetrazolium bromide (MTT, Sigma-Aldrich) assay, which is based on the ability of live cells to convert a tetrazolium salt into purple formazan. hUCB-MSCs (20,000 per well) were seeded in 24-well plates. After 48 hours of incubation, 50 ml MTT stock solution (5 mg/ml; Sigma) was added to each well, and the plates were further incubated for 4 hours at 37°C. The supernatant was removed, and 500 μl of DMSO was added to each well to solubilize the purple formazan crystals. The solution was then transferred to a 96-well microplate for measurement. The absorbance at a wavelength of 540 nm was measured using an EL800 microplate reader (BIO-TEK Instruments, Winooski, USA). All of the measurements were performed in triplicate.

### Western blot analysis

The cells were lysed with a protein lysis buffer (Pro-PREP, Intron Biotechnology, Korea) with a protease/phosphatase inhibitor cocktail, and protein concentration were determined using a DC assay kit (Bio-Rad, Berkeley, USA). The proteins were separated via 10-15% sodium dodecyl sulfate-polyacrylamide gel electrophoresis (SDS-PAGE), transferred to nitrocellulose membranes, blocked with 3% bovine serum albumin in Tris-buffered saline with Tween (TBST: 20 mM Tris-HCl [pH 7.6], 137 mM NaCl, 1% Tween 20), and probed with the indicated primary antibodies; rabbit anti-p16^INK4a^ (1:1000; Abcam, Cambridge, UK; ab108349), rabbit anti-BMI1 (1:1000, Cell Signaling Technology Europe, Leiden, The Netherlands; #6964s), mouse anti-GAPDH (1:3000, Millipore, Darmstadt, Germany; MAB374), rabbit anti-COX-2 (1:1000, Abcam; ab15191), rabbit anti-p-p38 (1:1000, Cell Signaling; #9211s), rabbit anti-MKP-1 (1:200, Santacruz, Texas, USA; sc-1102). The secondary antibodies were used according to the manufacturer's specifications; horseradish peroxidase (HRP)-conjugated antibodies (1:2000; Invitrogen, Carlsbad, USA; G21040, G21234) and binding was detected using an enhanced chemiluminescence (ECL) detection kit (Amersham Pharmacia Biotek, Amersham, UK).

### Isolation and culture of hUCB-derived MNCs

Human mononuclear cells were isolated from UCB samples. The UCB samples were mixed with HetaSep solution (StemCell Technologies, Canada) at a ratio of 5:1 and incubated at room temperature to the deplete erythrocyte portion. The supernatant was carefully collected and mononuclear cells were obtained using Lymphoprep^TM^ (StemCell Technologies, Vancouver, Canada) with density-gradient centrifugation at 2,500 rpm for 20 minutes. The isolated MNCs were washed twice in PBS and used for the co-culture experiments with hUCB-MSCs or were expanded in the supernatant media collected from cultured hUCB-MSCs.

### MNC proliferation assay

The isolated hUCB-MNCs were cultured in the supernatant media collected from normoxic- or hypoxic-cultured hUCB-MSCs. To investigate the role of BMI1 in the immunomodulatory properties of MSCs, the hUCB-MSCs were treated with 25 μg/ml of mitomycin C (from *Streptomyces caespitosus*) at 37°C for 1 hour. After washing twice in PBS, the cells were seeded in 96-well plates at 1×10^4^ cells/well and incubated for 24 hours. hMNCs were prepared as previously described, treated with concanavalin A (from *Canavalia ensiformis*) in RPMI medium and cultured at 1×10^5^ cells/well. After 3 days of co-culture, MNC proliferation was determined using a cell proliferation ELISA kit, (BrdU kit, Roche, Basel, Switzerland). Changes in absorbance (optical density, OD) in relation to the level of BrdU incorporation were measured spec-trophotometrically at 450 nm using a microplate reader.

### Cytokine production

Cells were treated with recombinant human IFN-γ and TNF-α (PeproTech, Rocky Hill, USA) for 24 hours, and PGE2 production was determined from the culture supernatant using a commercial ELISA kit (R&D Systems, Minneapolis, USA).

### Retroviral transduction

Viral production and transduction were performed as previously described (Yu et al., 2012). Briefly, the retroviral pMX-GFP, pMX-EZH2, pMX-SUZ12, pMX-BMI1, pSMP-Luc and pSMP-BMI1 plasmids were transfected into 293FT cells with VSV-G and gag/pol plasmids using the Fugene 6 transfection reagent (Roche). The viral supernatants were collected 48 and 72 hours post-transfection and were used to infect hUCB-MSCs in the presence of 5 μg/ml polybrene (Sigma). At 24 hours after transduction, the cells were washed more than four times in PBS and maintained in growth medium for expansion.

### Quantitative RT-PCR

Total RNA was extracted using a TRIzol kit (Invitrogen, USA) according to the manufacturer's protocol. 1 ug of RNA was reverse transcribed to cDNA using the Superscript First-Strand Synthesis System (Invitrogen, Carlsbad, USA). Relative mRNA levels were determined using the SYBR-Green PCR Master Mix (Applied Biosystems, Foster City, USA) with an ABI 7300 sequence detection system and the supplied software. Each gene expression level was normalized using GAPDH as the housekeeping control. At least three independent analyses were carried out for each gene.

### Chromatin immunoprecipitation

To demonstrate the binding of the BMI1 protein to the DUSP1 promoter, chromatin immunoprecipitation (ChIP) assays were carried out according to the manufacturer's instructions (ChIP assay kit, Millipore, Darmstadt, Germany). Briefly, 1 to 2×10^7^ cells were cross-linked with 1% formaldehyde for 10 minutes at 37°C and then sub-sequently harvested and washed with ice-cold PBS containing protease inhibitors. The cell lysates were incubated with the BMI1 antibodies. Immune complexes were precipitated and the promoter regions of DUSP1 and p16^INK4a^ were amplified via PCR or quantitative PCR using the primers spanning the potential DUSP1 binding site.

## SUPPLEMENTAL DATA FIGURES AND TABLE


