# Supplementary material for: BMI1 inhibits senescence and enhances the immunomodulatory properties of human mesenchymal stem cells via the direct suppression of MKP-1/DUSP1
Source: Aging (Albany NY). 2016 Jul 22;8(8):1670–85. doi: 10.18632/aging.101000 (PMC5032689; doi:10.18632/aging.101000)
Supplement: Supplementary file 1 [file aging-08-1670-s001.pdf]

SUPPLEMENTARY DATA

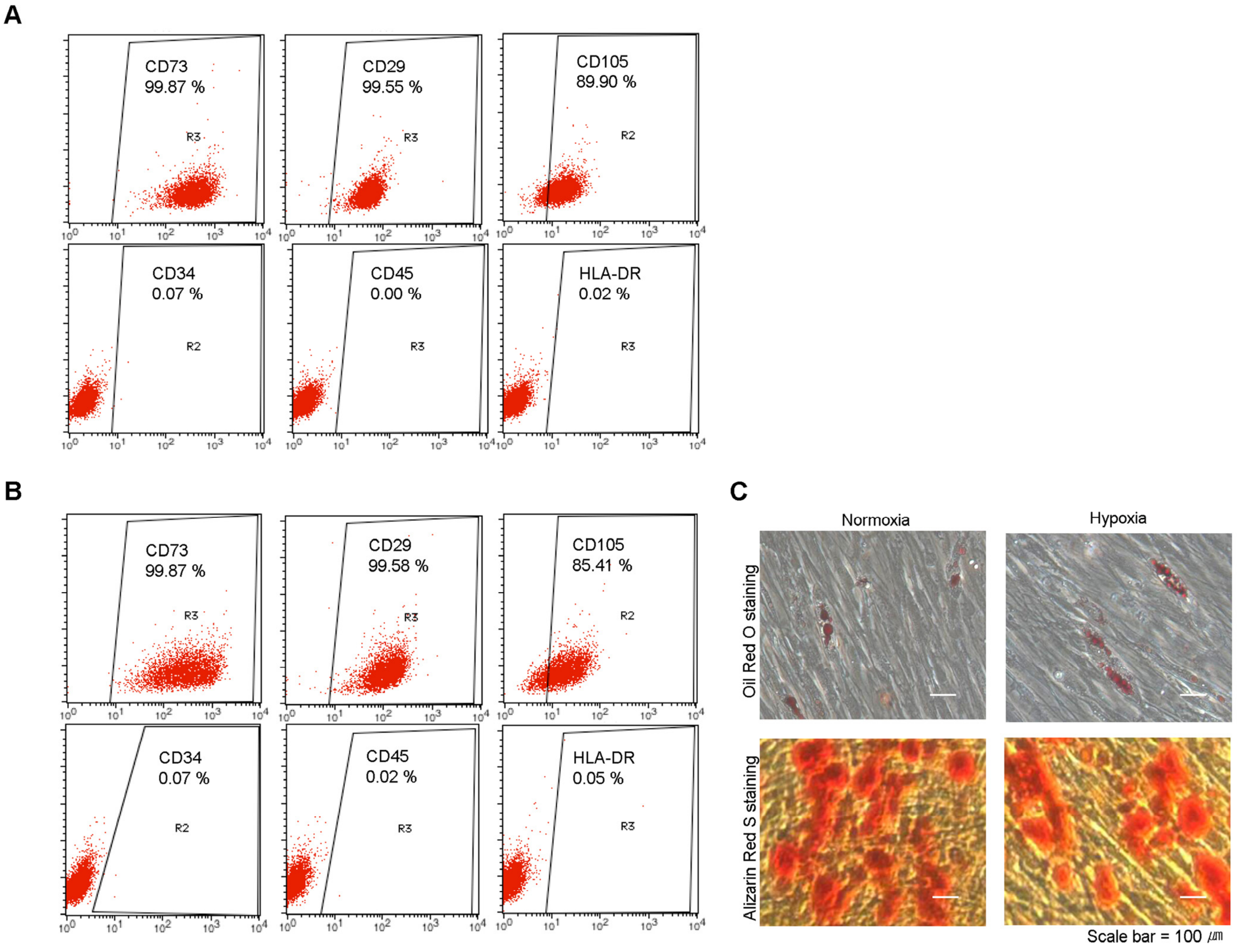

Supplementary Figure S1. Characterization of hUCB-MSCs cultured in normoxia and hypoxia. Normoxic (**A**)- and hypoxic (**B**)-cultured hUCB-MSCs (1×10^6 cells/ml) were stained with FITC- or PE-conjugated antibodies specific for human CD29, CD34, CD45, CD73, CD105, and HLA-DR. (**C**) Images of differentiated hUCB-MSCs after induction into specific tissues. Lipid droplet accumulation in differentiated cells was visualized using Oil Red O staining after 2.5 weeks of adipogenic induction. Calcium deposits were stained with Alizarin Red S after 2.5 weeks of osteogenic induction. The results show 1 representative sample from of 3 independent experiments.

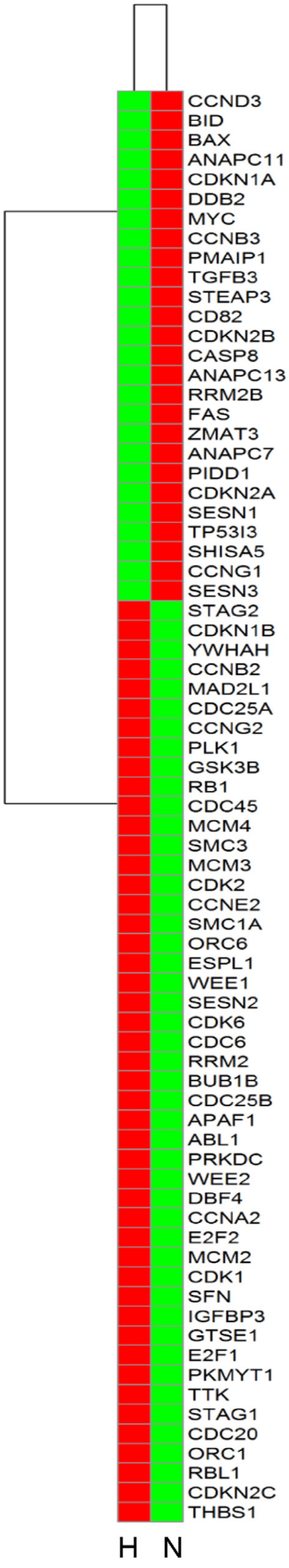

Group	Gene	Annotation
1	E2F2	E2F transcription factor 2
1	SFN	stratifin
1	SESN2	sestrin 2
1	CDC20	cell division cycle 20
1	CDKN2C	cyclin-dependent kinase inhibitor 2C (p18, inhibits CDK4)
1	ORC1	origin recognition complex, subunit 1
1	RRM2	ribonucleotide reductase M2
1	MCM2	minichromosome maintenance complex component 2
1	STAG1	stromal antigen 1
1	CCNA2	cyclin A2
1	TTK	TTK protein kinase
1	IGFBP3	insulin-like growth factor binding protein 3
1	DBF4	DBF4 zinc finger
1	CDK6	cyclin-dependent kinase 6
1	WEE2	WEE1 homolog 2 (S. pombe)
1	PRKDC	protein kinase, DNA-activated, catalytic polypeptide
1	ABL1	ABL proto-oncogene 1, non-receptor tyrosine kinase
1	CDK1	cyclin-dependent kinase 1
1	WEE1	WEE1 G2 checkpoint kinase
1	ESPL1	extra spindle pole bodies homolog 1 (S. cerevisiae)
1	APAF1	apoptotic peptidase activating factor 1
1	THBS1	thrombospondin 1
1	BUB1B	BUB1 mitotic checkpoint serine/threonine kinase B
1	PKMYT1	protein kinase, membrane associated tyrosine/threonine 1
1	ORC6	origin recognition complex, subunit 6
1	CDC6	cell division cycle 6
1	CDC25B	cell division cycle 25B
1	E2F1	E2F transcription factor 1
1	RBL1	retinoblastoma-like 1
1	GTSE1	G-2 and S-phase expressed 1
1	SMC1A	structural maintenance of chromosomes 1A
2	TP53I3	tumor protein p53 inducible protein 3
2	STEAP3	STEAP family member 3, metalloreductase
2	CASP8	caspase 8, apoptosis-related cysteine peptidase
2	SHISA5	shisa family member 5
2	ANAPC13	anaphase promoting complex subunit 13
2	ZMAT3	zinc finger, matrin-type 3
2	CCNG1	cyclin G1
2	SESN1	sestrin 1
2	RRM2B	ribonucleotide reductase M2 B (TP53 inducible)
2	CDKN2A	cyclin-dependent kinase inhibitor 2A
2	CDKN2B	cyclin-dependent kinase inhibitor 2B (p15, inhibits CDK4)
2	FAS	Fas cell surface death receptor
2	PIDD1	p53-induced death domain protein 1
2	CD82	CD82 molecule
2	SESN3	sestrin 3
2	ANAPC7	anaphase promoting complex subunit 7
2	TGFB3	transforming growth factor, beta 3
2	PMAIP1	phorbol-12-myristate-13-acetate-induced protein 1
3	CDC25A	cell division cycle 25A
3	GSK3B	glycogen synthase kinase 3 beta
3	CCNG2	cyclin G2
3	MAD2L1	MAD2 mitotic arrest deficient-like 1 (yeast)
3	MCM4	minichromosome maintenance complex component 4
3	SMC3	structural maintenance of chromosomes 3
3	CDKN1B	cyclin-dependent kinase inhibitor 1B (p27, Kip1)
3	RB1	retinoblastoma 1
3	CCNB2	cyclin B2
3	PLK1	polo-like kinase 1
3	CDC45	cell division cycle 45
3	YWHAH	tyrosine 3-monooxygenase
4	CDKN1A	cyclin-dependent kinase inhibitor 1A (p21, Cip1)
4	DDB2	damage-specific DNA binding protein 2, 48kDa
4	ANAPC11	anaphase promoting complex subunit 11
4	BAX	BCL2-associated X protein
4	BID	BH3 interacting domain death agonist
5	CCND3	cyclin D3
6	MCM3	minichromosome maintenance complex component 3
6	CDK2	cyclin-dependent kinase 2
7	CCNE2	cyclin E2
8	MYC	v-myc avian myelocytomatosis viral oncogene homolog
8	CCNB3	cyclin B3
9	STAG2	stromal antigen 2

Supplementary Figure S2. Hierarchical clustering analysis of normoxic- and hypoxic-cultured hUCB-MSCs.

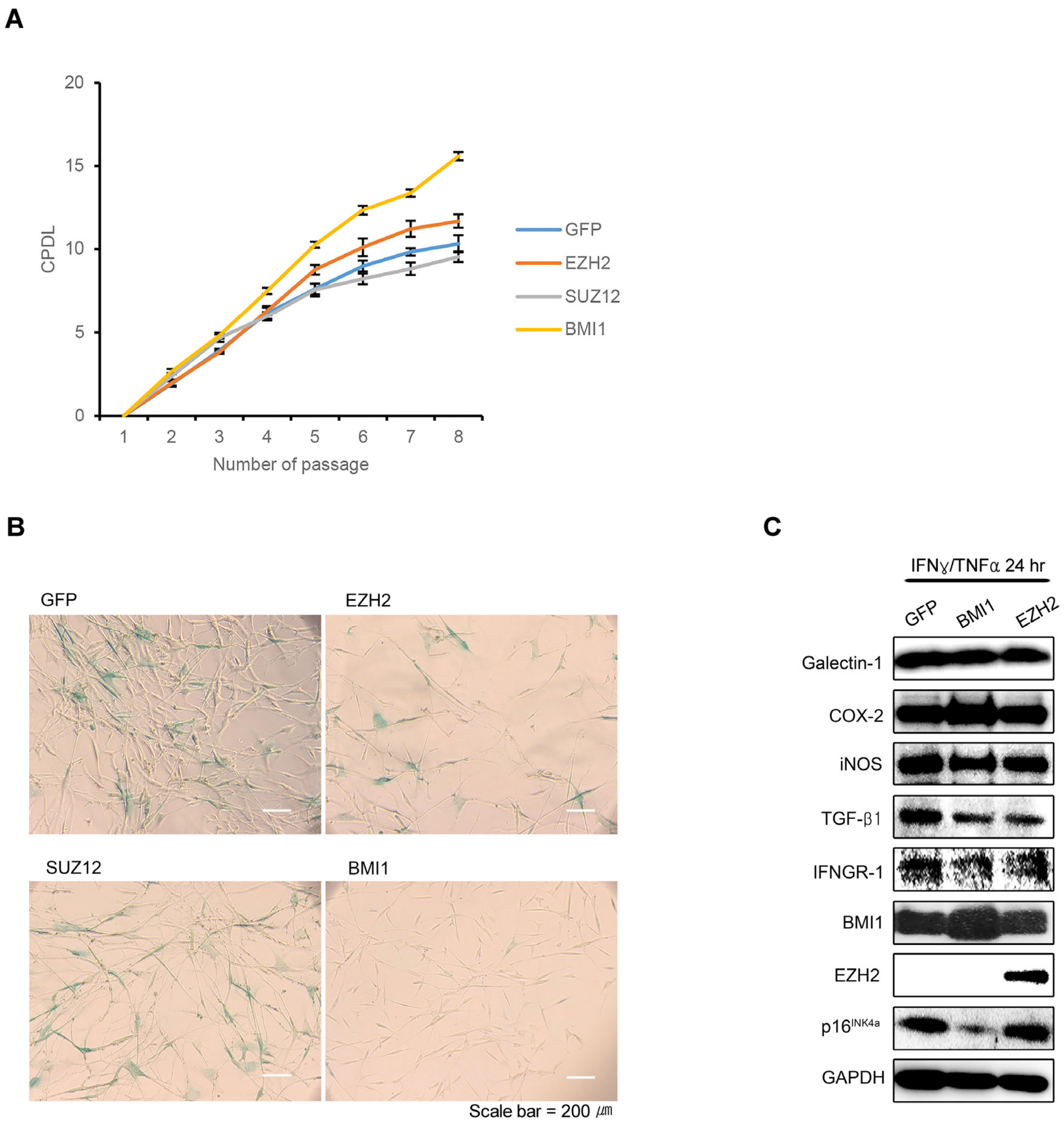

Supplementary Figure S3. Among the polycomb group proteins, BMI1 most effectively inhibits cellular senescence and improves the expression of COX-2. (**A**) After the overexpression of GFP, EZH2, SUZ12 and BMI1, hUCB-MSCs were subcultured and CPDLs were calculated. (**B**) After several passages, SA-β-gal staining was conducted to assess the senescent state of hUCB-MSCs overexpressing each factor. (**C**) GFP-, BMI1- and EZH2-transduced hUCB-MSCs were treated with IFN-γ and TNF-α for 24 hours, and western blot analyses were conducted to investigate the effects of the immunomodulatory proteins and COX-2 expression.

Table S1. Pimers used in qRT-PCR and ChIP assay

qPCR Primers used for mRNA expression analysis

Target	Forward	Reverse
BMI1	GACTCTGGGAGTGACAAGGC	AGATTGGTGGTTACCGCTGG
p16INK4a	GAAGGTCCCTCAGACATCCC	CCCTGTAGGACCTTCGGTGA
DUSP1	CTGCCCTTTCTGTACCTGGG	GGTTGTCCTCCACAGGGATG
GAPDH	TGATGACATCAAGAAGGTGGTG	ACCCTGTTGCTGTAGCCAAAT

qPCR Primers used for ChIP analysis

Location	Forward	Reverse
p16INK4a promoter (-38 ~ -158)	GCACTCAAACACGCCTTTGC	AGAGCCAGCGTTGGCAAGGA
DUSP1 promoter 1 (-1815 ~ -1717)	GCGCCCAGCTCTTAAAAAGT	CCGACTTGATTTGTCCCATT
DUSP1 promoter 2 (-662 ~ -543)	GCTCGAGTCGGTCTTGGTAG	GACTTGCCCAGAACCACACT
DUSP1 promoter 3 (-106 ~ -16)	CCGTCACGTGATCACCATT	GCGTTTATATGCGGCCTCT
